# Evidence of heat-resistant microorganisms with a special emphasis on filamentous Actinomycetes in hyper-arid soils of Gandom Beryan area, Lut Desert, Iran

**Published:** 2017-12

**Authors:** Somaye Mazkour, Saeid Hosseinzadeh, Seyed Shahram Shekarforoush

**Affiliations:** Department of Food Hygiene and Public Health, School of Veterinary Medicine, Shiraz University, Shiraz, Iran

**Keywords:** Lut Desert, Heat-resistant microorganism, filamentous Actinomycetes

## Abstract

**Background and Objectives:**

In the present study, the Lut Desert, Iran was chosen as one of the hottest places in the world (with the recorded temperature of 70.7°C during 2003–2009) to find out whether any heat-resistant microorganisms were present in the soil.

**Materials and Methods:**

The samples were collected from surface and depth of three identified places of Gandom Beryan in the Lut Desert. Chemical analysis and enumeration of the total bacteria, yeasts and molds were performed. Four selective culture media were employed to isolate the filamentous actinomycetes. The suspected colonies were further confirmed using PCR assay. Then the culture cell-free-supernatants (CFS) of isolates were used to investigate their antimicrobial activity against *Staphylococcus aureus, Bacillus cereus, Salmonella* Typhimurium and *Escherichia coli*.

**Results:**

Chemical analysis of the samples included moisture (0.2–0.9%), ash (85–91%), organic materials (8.3–14.4%), pH (7.59–9.40) and electrical conductivity (380–2000 μS/cm). The number of isolated bacteria and molds varied from 0–20 to 0–40 CFU/g, respectively. Number of Actinomycetes isolated from the soil samples were between 0–12.2 CFU/g. Nine isolated colonies were identified as filamentous Actinomycetes. To determine the possibility of antimicrobial peptides, the CFS (cell-free supernatant) was firstly neutralized by NaOH and catalase. The results showed that none of the CFS of the isolates was effective against *E. coli, S.* Typhimurium and *S. aureus*, while the maximum inhibitory effect was investigated on *B. cereus*, which was 33.1%±1.19% (mean ± SD).

**Conclusion:**

The results of the current study imply the presence of rare heat-resistant microorganisms in the soil of Gandom Beryan which may be further used to find out more about the function of natural bioactive compounds. Actinomycetes, as extremophile microorganisms, have shown the greatest genomic and metabolic diversity, as such the discovery of the novel Actinomycetes as a source of secondary metabolites is essential.

## INTRODUCTION

Lut Desert, located in the Southeast of Kerman, Iran is the twenty-seventh desert of the world. In 2005, Lut Desert (29.9 N, 59.1 E) with the temperature 70.7°C has been recorded as the hottest place on the earth. This was the highest temperature recorded during 2003–2009 (1). In fact, it has been said that Lut Desert is so inhospitable that even bacteria cannot survive. But maybe due to some reasons, including climate and special chemical properties it could be a source of novel strain of microorganisms (2–6). One of the most important groups is Actinomycetes which are mainly found in soil under various climatic conditions (7, 8). Phenotypically, they are Gram positive bacteria with some filamentous species resembling the mold. They produce spores and also direct or spiral aerial mycelium (9) Actinomycetes, especially streptomycetes, constitute the most abundant source of natural products, notably antibiotics (2, 10, 11), antitumor compounds (12) and immunosuppressive agents (13). There are more than 22,000 known microbial secondary metabolites, 70% of which are produced by Actinomycetes (14). It is predicted that only a few of all antibiotics has been discovered and finding the remaining needs more researches. Several studies have been conducted in recent years in order to find new Actinomycetes producing wide variety of new metabolites from untapped areas. One of the most important untapped areas is hot and dry deserts (8, 15). The Lut Desert is an untapped habitat on the earth that can be a place to look for the novel heat-resistant Actinomycetes due to hyper-arid condition and high temperature. The aim of this study was to investigate the chemical and microbiological properties of soil of the Lut Desert as a source of Actinomycetes.

## MATERIALS AND METHODS

### Sample collection.

**Fig. 1.**
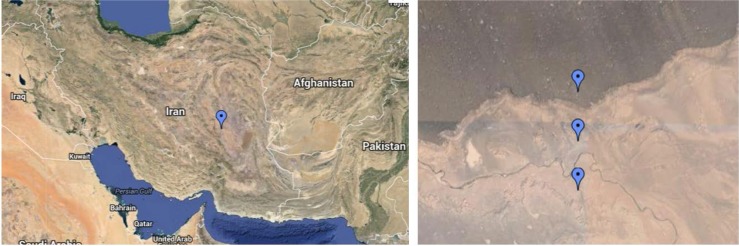
Sampling locations of the Gandom Beryan region in the Lut Desert. The exact longitude of the area was 57.67 E and the latitudes were 31.02 N (1), 31.01 N (2) and 31.00 N (3).

In July 2012, forty six soil samples were collected from three identified places in Gandom Beryan region in the Lut Desert. Longitude of the area was 57.67 E and the latitudes were 31.02 N, 31.01 N and 31.00 N, respectively (Fig. 1). At each location, the samples were taken at 10 m intervals; one sample was collected from the surface to a depth of 5 cm and the others from a depth of 5 to 20 cm of soil, using a sterile scoop. The samples were transferred on ice to the lab and stored at −20°C until further analysis.

### Chemical analysis.

The pH and electrical conductivity of the samples were determined. The values were measured using a standardized electrode attached to a digital pH meter (CG824, Germany) and electro conductivity meter (HANNA instruments, USA), respectively.

Moisture and ash were determined according to recommended methods (16). Briefly, the moisture (%, w/w) was determined by drying 10 g of the sample at 105°C to constant weight and then ash contents were performed at 700°C for 1h (%, w/w). All parameters were measured in triplicate.

### Enumeration of the fungi and bacteria.

After properly mixing the soil sample, a 1:1 (W/W) suspension in sterile saline solution was prepared (1/2 dilution). The suspension was shaken for 10 min at 37°C. In order to make 1/20 dilution, 1 ml of the soil suspension was added to 9 ml sterile saline solution. For an enumeration of fungi and bacteria 0.1 ml of the 1/2 and 1/20 dilutions were spread over the surfaces of potato dextrose agar (Merck, Germany) containing 200 μg/ml chloramphenicol and nutrient agar (Merck, Germany) containing 40 μg/ml acriflavin and 150 μg/ml nystatin, respectively. Potato dextrose agar was incubated at 20°C for 7 days and nutrient agar was incubated at 35°C for 5 days. Number of colonies on each media was counted and expressed as CFU/g of soil. All measurements were performed in duplicate.

### Isolation of Actinomycetes.

Isolation of Actinomycetes was carried out after inactivating the vegetative bacteria in the suspension (1/2 dilution) by heating at 55°C for 6 min (6), which was subsequently sub-cultured onto four selective medias including: humic acid-vitamin agar containing 50 μg/ml cyclohexamide and 10 μg/ml nalidixic acid (17), starch-casein agar containing 25 μg/ml cyclohexamide and 25 μg/ml nystatin (18), raffinose-histidine agar containing 25 μg/ml cyclohexamide and 25 μg/ml nystatin (19) and glucose-yeast extract agar containing 20 μg/ml rifampicin (20). The selective culture media were incubated at 28°C for 3 weeks. The number of Actinomycetes on each selective media was counted after Gram staining of all the suspected colonies. The pure colonies were then sub-cultured on humic acid-vita-min agar without any antibiotic supplements. After 10 days of incubation at 28°C, one pure colony was inoculated into tryptic soy broth at 28°C for 72 h, followed by DNA extraction for PCR amplification. Total genomic DNA was extracted using CinnaGen kit (Tehran, Iran). The PCR assay was performed in a final volume of 25 μl, containing 2.5 μl PCR buffer, 2.5 mM MgCl_2_, 200 mM dNTP, 400 μM of each primers (243F 5-GGATGAGCCCGCGGCCTA-3, A3R 5-CCAGCCCCACCTTC GAC-3) (21), 1.5 U of Taq DNA polymerase and 2 μl extracted DNA. Samples were amplified as follows: 95°C for 10 min, followed by 30 cycles of 94°C for 45 sec, 68°C for 2 min and 72°C for 1 min, with a final extension at 72°C for 10 min. The PCR products were finally electrophoresed on a 1.5% ethidium bromide-stained agarose gel and the specific DNA fragments with desired size of 1.25 kb was observed.

### Antimicrobial activity of isolated Actinomycetes.

Culture cell-free-supernatants (CFS) of selected isolates were obtained from the inoculated TSB broth, at 28°C for 72 h after centrifugation at 3000 × g for 5 min. The well diffusion assay was performed to investigate the antimicrobial activities of the neutralized CFSs (pH 7.0) on *S. aureus* (ATCC 6538), *B. cereus* (ATCC 14579), *S.* Typhimurium (ATCC 14028) and *E. coil* (ATCC 35218) (22). The antimicrobial activity of the effective neutralized CFS was also detected after decomposition of possible hydrogen peroxide. 150 IU/ml catalase (Sigma-Aldrich, USA) incubated with CSF at 25°C for 2 h was used. The enzyme was then inactivated by heating at 65°C for 2 h (23). The well diffusion assay was performed again and the effective ones were treated with trypsin, pepsin, proteinase K and a-chymotrypsin (Merck, Germany) to a final concentration of 1 mg/ml in 0.5 mM Tris–0.2 mM CaCl_2_ buffer (pH 8.5). Samples were incubated at 37°C for 1 h. The enzyme was then inactivated by heating at 65°C for 2 h (23). It was followed by another well diffusion assay.

For measurement of the growth inhibitory effect of CFSs, the percentage of inhibition was calculated as follows (24):

% growth inhibition= [(O – E)/O] × 100

Where O is (OD of the positive control at 24 h - OD of the positive control at 0 h) and E is (OD of the organism in the presence of CFS at 24 h - OD of its at 0 h).

### Statistical analysis.

The results were analyzed using one-way analysis of variance and the statistical significance of differences between mean values was analyzed by Duncan’s multiple range tests. However, non-parametric data were analyzed using MacNemar test. P-values less than 0.05 were considered statistically significant. Analysis was performed using Statistical Package for Social Sciences (SPSS) software (SPSS 16 for windows, SPSS Inc, Chicago, IL, USA).

## RESULTS

### Chemical analysis.

Humidity of the soil samples was recorded 0.35 - 0.70%. The moisture of surface samples of the location one was significantly lower than others (*p* < 0.05). Surface samples revealed almost the same organic matter. Details of the chemical analysis are shown in Table 1.

**Table 1.** Chemical analysis of the soil samples of Gandom Beryan region, the Lut Desert, Iran.

Location of sampling*	**Depth of sampling**	**Number of samples**	**Moisture content (%)**	**Ash (%)**	**Organic matter (%)**	**pH**	**Electrical conductivity (μS/cm)**
1	S	10	0.35±0.14^a^	87.9±1.1^abc^	11.6±1.2^a^	8.7±0.4^a^	546.3±132.4^a^
	D	10	0.54±0.17^b^	88.5±1.6^bc^	11.0±1.5^ab^	8.6±0.3^a^	636.2±142.9^ab^
2	S	7	0.56±0.18^b^	87.6±1.4^ab^	11.9±1.4^a^	8.5±0.2^b^	712.4±101.0^bc^
	D	7	0.66±0.13^b^	89.3±1.5^c^	10.1±1.5^b^	8.5±0.3^b^	819.6±78.2^c^
3	S	6	0.68±0.15^b^	86.7±1.4^a^	12.7±1.4^a^	8.5±0.6^a^	1921.5±96.7^d^
	D	6	0.70±0.18^b^	87.7±1.2^ab^	11.6±1.2^a^	8.1±0.3^b^	1953.0±54.1^d^

* Longitude of sampling locations was 57.67 E and the latitudes were 31.02 N, 31.01 N and 31.00 N, respectively. Values are mean ± standard deviation. S: surface to a depth of 5 cm, D: the depth of 5 to 20 cm. The different letters in columns indicate significant differences (*p* < 0.05).

### Total count of bacteria and fungi.

In this study, 46 soil samples from three regions of the Lut Desert were evaluated for total count of bacteria and fungi. Generally, the minimum and the maximum number of bacteria were respectively 0 and 20 CFU/g and the number of mold was variable (0–40 CFU/g). Total count of bacteria and molds are shown in Table 2. No bacteria were found in soil samples of location 2 and 3 and also in the soil depth of location 1.

**Table 2.** Total counts of bacteria and fungi of the soil samples of Gandom Beryan region, Lut Desert, Iran.

Location of sampling*	**Depth of sampling**	**Number of Samples**	**Bacteria (CFU/g)**	**Fungi (CFU/g)**
1	S	10	3.0±2.1	5.0±1.7^a^
	D	10	0	16.0±3.7^b^
2	S	7	0	24.3±3.0^b^
	D	7	0	18.6±2.6^b^
3	S	6	0	18.3±4.8^b^
	D	6	0	15.0±3.4^b^

* Longitude of sampling locations was 57.67 E and the latitudes were 31.02 N, 31.01 N and 31.00 N, respectively. Values are mean ± standard deviation. S: surface to a depth of 5 cm, D: the depth of 5 to 20 cm. The different letters indicate significant differences (*p* < 0.05).

### Isolation of Actinomycetes.

**Fig. 2.**
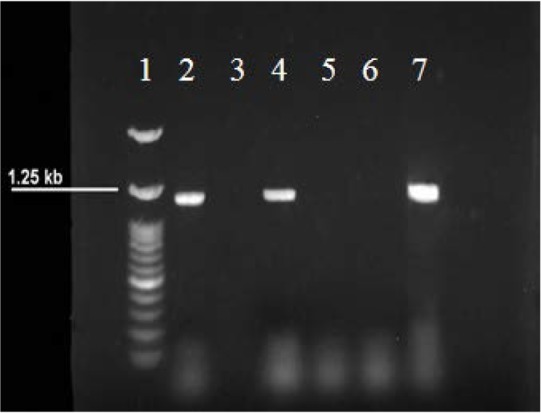
Representing 1.5% agarose gel electrophoresis of the PCR products of the isolated Actinomycete. Lane 1: 100 bp DNA marker; Lane 2: positive control (genus Streptomyces); Lane 3: negative control (no template); Lane 5 & 6: negative samples; Lane 4 & 7: 1250 bp amplicon corresponding to the positive samples (Actinomycetes).

For isolation of Actinomycetes, all samples were cultured on four selective culture media. Number of Actinomycetes on selective media is shown in Table 3. Forty-four Actinomycetes were isolated from surface and the depth. Out of 44 Actinomycetes isolates, only 9 species were confirmed by PCR (Fig. 2).

**Table 3.** Number of Actinomycetes on selective media of the soil samples of Gandom Beryan region, Lut Desert, Iran.

Location of sampling*	**Depth of sampling**	**Number of Samples**	**humic acid-vitamin agar (CFU/g)**	**starch-casein agar (CFU/g)**	**raffinose-histidine agar (CFU/g)**	**glucose-yeast extract agar (CFU/g)**
1	S	10	8.5±6.3^ab^	5.5±9.6	5.0±1.6^b^	4±4.6^b^
	D	10	7.5±7.0^ab^	9.5±12.6	1.5±2.4^ab^	1.0±3.2^ab^
2	S	7	8.5±13.6^b^	6.4±11.1	0^a^	0.7±1.9^ab^
	D	7	12.2±8.6^ab^	3.6±4.8	0^a^	0.7±1.9^ab^
3	S	6	3.3±8.2^a^	4.2±8.0	1.7±2.6^ab^	0^a^
	D	6	5.8±5.8^ab^	1.7±2.6	0.8±2.0^ab^	1.7±2.6^ab^

* Longitude of sampling locations was 57.67 E and the latitudes were 31.02 N, 31.01 N and 31.00 N, respectively. Values are mean ± standard error. S: surface to a depth of 5 cm, D: the depth of 5 to 20 cm. The different letters in columns indicate significant differences (*p* < 0.05).

### Antimicrobial activity of the isolates.

Table 4 shows the percentage of growth inhibition of CFS of isolated Actinomycetes against four pathogenic bacteria. None of the neutralized cell-free supernatant of the isolates was effective against *E. coli*, whereas one and six of them inhibited the growth of *S.* Typhimurium and *S. aureus*, respectively. Six isolates were also influenced the growth of *B. cereus*. After using catalase enzyme, just four isolates inhibited the growth of *B. cereus* and among these four isolates, just one of them still had antimicrobial effect after using protease. This suggests the influence of the specific peptides. It seems that they are able to produce peptides with antimicrobial activity.

**Table 4.** Percentage of growth inhibition of Actinomycees from soil samples of Gandom Beryan region, Lut Desert, Iran against four pathogenic bacteria.

Isolated Actinomycetes	Challenged bacteria							

	*E. coli*		*S.* Typhimurium		*S. aureus*		*Bacillus cereus*	
			
	After step 1	After step 2	After step 1	After step 2	After step 1	After step 2	After step 1	After step 2
1	0	-	0	-	14.3±2.0	0	33.9±2.6	34.0±2.7
2	0	-	0	-	36.1±4	0	0	-
3	0	-	0	-	25.6±3.5	0	33.9±2.6	36.4±3.1
4	0	-	0	-	0	-	20.6±1.9	0
5	0	-	0	-	0	-	0	-
6	0	-	0	-	0	-	33.3±3.0	34.1±1.6
7	0	-	0	-	36.9±3.8	0	23.1±2.2	0
8	0	-	16.5±2.7	0	16.1±2.4	0	0	-
9	0	-	0	-	16.1±2.4	0	19.5±2.7	20.6±2.7

Percentage of growth inhibition of culture cell-free supernatant (CFS) are after neutralization of acidity (Step 1) and decomposition of H_2_O_2_ (Step 2) at 37°C.

Values are mean ± standard deviation of 8 replicates.

## DISCUSSION

Average soil moisture obtained from the surface of the first region was significantly lower, which can be attributed to greater energy intake. The first area has a higher altitude than the other regions and was covered entirely by black stones. The average number of fungi obtained in the present study showed a direct correlation with soil moisture (r = 0.44, *p* = 0.002). The organic matter obtained from the Atacama Desert was 0.02 to 0.03%, that was much less than organic matter of the Lut Desert (6). Our results showed the range of 0.55–1.95 mS/cm for the electrical conductivity which was near to the results from Atacama Desert. The electrical conductivity of the Atacama Desert soil has been reported between 0.01–0.35 (26), 0.15–1.77 (27) and 2.49 mS/cm (28). The more altitude we took soil sample, the more electrical conductivity was obtained. The obtained pHs in Atacama Desert were lower than the pHs recorded in the current study. For example, the pH value obtained from the depth of 25–30 cm was 7.01–7.54 (20), from the surface samples it was 6.8–7.3 (28, 29), from other surface samples it was 7.6 to 7.7 (6) and samples obtained from different places were 7.9, 8 and 8.1 (27). Rin Bagaly examined different depth (0–90 cm) of desert soil and the pH was obtained 7.16 to 8.27 (30). The pH of soil is one of the major factors affecting the growth of microorganisms, because most of the bacteria and fungi are able to grow at the nutrient pH (31). The pH from deserts is generally higher than the forests. The pH of the dry sites of forest regions is 5.4 (depth of 10 cm) and 5.59 (depth of 10 to 20 cm) and in the wet sites was respectively 5.56, 5.69 (25).

In the present study, total bacterial count in most samples was 0 CFU/g which was quite similar to the findings of Okoro et al. (6). It seems that drying of hyper-arid region is the main inhibiting factor of the growth and survival of bacteria. Rin Bagaly examined four different depths (from the surface to 90 cm), the total bacterial count of deep regions was between 0 and 8.07×10^3^ CFU/g and the surface total count was less (30). They believe that the hyper-arid region of the Atacama Desert is an analog of Mars, both on the surface and in the subsurface.

There are reports indicating Actinomycetes have been isolated from the Sahara Desert soil, the Sonoran Desert and also Indian Thar Desert (3–5, 15). Kurapova et al. expressed that thermotolerant and thermophilic Actinomycetes were found in high abundance in the Mongolian Desert soils (32).The deserts represent the dry limit of microbial life on the planet (33, 34), however; Actinomycetes are widely distributed in marine and terrestrial habitats, especially in soil (8, 35). Lut Desert is an untapped region on the earth that can be a place to look for the novel strains of Actinomycetes due to hyper-arid condition and high temperature.

The maximum inhibitory effect of CFS of isolated Actinomycetes on the growing of *B. cereus* was 33.1±1.19%. Hozzein et al. isolated Actinomycets from Egyptian Desert that 43.75% of the active isolates have activity against Gram-positive bacteria only, 28.13% have activity against both Gram-positive and Gram-negative bacteria (36). Matsumoto and Takahashi introduced endophytic Actinomycetes as a new source of novel compounds (37). Azerang and Sardari mentioned genus *Streptomyces*, Actinomycetes as the most important source for various antibacterial pharmaceutical agents (38).

As a final conclusion, the Lut Desert is strongly recommended as an untapped place in the world to find novel strains of microorganisms as a potent source of novel antibiotics. Considering the previous works, we focused on the active isolate from Lut Desert in order to suggest novel antimicrobial compounds.
